# Spatial Reversal Learning in Chronically Sensitized Rats and in Undrugged Sensitized Rats with Dopamine D2-Like Receptor Agonist Quinpirole

**DOI:** 10.3389/fnbeh.2014.00122

**Published:** 2014-04-11

**Authors:** Hana Hatalova, Dominika Radostova, Adela Pistikova, Karel Vales, Ales Stuchlik

**Affiliations:** ^1^Institute of Physiology, Academy of Sciences of the Czech Republic, Prague, Czech Republic

**Keywords:** reversal, flexibility, cognitive coordination, quinpirole, behavior, rat, obsessive–compulsive disorder

## Abstract

Dopamine plays a role in generating flexible adaptive responses in changing environments. Chronic administration of D2-like agonist quinpirole (QNP) induces behavioral sensitization and stereotypical behaviors reminiscent of obsessive–compulsive disorder (OCD). Some of these symptoms persist even after QNP discontinuation. In QNP-sensitization, perseverative behavior has often been implicated. To test the effect of QNP-sensitization on reversal learning and its association with perseveration we selected an aversively motivated hippocampus-dependent task, active place avoidance on a Carousel. Performance was measured as the number of entrances into a to-be-avoided sector (errors). We tested separately QNP-sensitized rats in QNP-drugged and QNP-undrugged state in acquisition and reversal tasks on the Carousel. In acquisition learning there were no significant differences between groups and their respective controls. In reversal, QNP-sensitized drugged rats showed a robust but transient increase in number of errors compared to controls. QNP-sensitized rats in an undrugged state were not overtly different from the control animals but displayed an altered learning manifested by more errors at the beginning compensated by quicker learning in the second session compared to control animals. Importantly, performance was not associated with perseveration in neither QNP-sensitized drugged nor QNP-sensitized undrugged animals. The present results show that chronic QNP treatment induces robust reversal learning deficit only when the substance is continuously administered, and suggest that QNP animal model of OCD is also feasible model of cognitive alterations in this disorder.

## Introduction

Cognitive flexibility is an ability to detect a shift in stimulus–feedback contingencies. It requires the recognition of the irrelevance of a response and response to a new stimulus/reward contingency. Due to its relevance to many psychiatric conditions including schizophrenia (Pantelis et al., 1999; Morris, 2013), obsessive–compulsive spectrum disorders (OCD) (Chamberlain et al., 2005), substance abuse (Jentsch et al., 2002; Ersche et al., 2008, 2011), autism (Yerys et al., 2009), and due to its necessity for successful functioning of every organism in a changing environment – cognitive flexibility is an intensively studied cognitive domain. One common task to assess cognitive flexibility is reversal learning (Klanker et al., 2013). Reversal learning has also been recently proposed to mimic some aspects of compulsivity (Homberg, 2013).

It has been proposed that dopamine plays an important role in reversal learning via dopamine D2-like receptor signaling. Systemic D2/D3 antagonist raclopride impaired reversal learning, while D1/D5 antagonist SCH 23390 did not (Lee et al., 2007). A blockade of D2-like receptors in the prefrontal cortex (PFC) was associated with a pronounced perseverative deficit in a set-shifting task (Floresco and Grace, 2003) and an activation of D2-like receptors in the nucleus accumbens impeded maintaining novel stimulus–reward contingencies (Haluk and Floresco, 2009). There is compelling evidence of D2-like signaling in striatal regions being essentially involved in reversal learning (Haluk and Floresco, 2009; Clarke et al., 2011; Groman et al., 2011); yet compared to antagonizing D2-like receptors very few studies have focused on effect of selective stimulation of D2-like receptors. For example, only one study has focused on the effect of systemic application of quinpirole (QNP), a D2-like agonist on reversal learning (Boulougouris et al., 2009). Boulougouris and colleagues showed that QNP produces a perseverative reversal learning deficit after acute systemic administration without a deficit in acquisition learning. This effect was attributed to D2-like receptor stimulation because concurrent antagonizing of D3 receptors by nafadotride did not ameliorate the effect, while D2/D3 antagonist raclopride did have this effect.

Repeated QNP administration produces escalated behavioral effects of acute QNP administration. Similarly to other stimulants it produces hyperlocomotion (Mattingly et al., 1993). In addition, QNP-treated rats also display environment-dependent perseveration in a spontaneous alternation task (Einat and Szechtman, 1995). QNP-sensitization is not associated with stereotypy of body movements such as after application of amphetamine (Wolgin, 2012), but only with path stereotypy and checking in an enriched open-field (Szechtman et al., 1998). Based on the striking similarity between QNP-sensitized behavior in rats and obsessive–compulsive symptoms in humans, it was proposed that sensitization with QNP may serve as a useful rat model of OCD. Co-administration of the tricyclic antidepressant clomipramine, effective in ameliorating symptoms in the treatment of OCD (Piccinelli et al., 1995), adds to the predictive validity of QNP-sensitization as a rat model of OCD (Szechtman et al., 1998). Additionally, the behavioral effects of chronic QNP administration are considered also to mimic some of behavioral characteristics of schizophrenia, specifically psychotic polydipsia (Goldman et al., 1988; De Carolis et al., 2010, 2011; Milella et al., 2010).

Prolonged QNP treatment was associated with changes in CNS but very little is known about the behavioral effects after QNP treatment is terminated. Sensitization by QNP alters dopamine levels in the *substantia nigra, striatum*, and the PFC (Sullivan et al., 1998) and alters D2 and D3 receptor binding in the *nucleus accumbens*, ventral *pallidum*, and *substantia nigra* (Stanwood et al., 2000). Based on these wide scale alterations in the dopamine system, we expect that these alterations manifest themselves on a behavioral level as well. Indeed, some QNP-specific behaviors of sensitized drugged rats such as perseveration and conservativeness of travel routes are also observed in sensitized undrugged rats, albeit to a lesser extent (Einat and Szechtman, 1993). No change in reversal learning performance or locomotion – alterations, which are observed in drugged rats – was detected in this study.

The present study employed active place avoidance on a Carousel [also known as active allothetic place avoidance; AAPA; (Bures et al., 1997; Petrasek et al., 2013; for review, see Stuchlík et al., 2013)]. This task is a hippocampus-dependent spatial task originally developed in our laboratory to study higher-order spatial navigation and cognitive coordination and was shown to be sensitive in the detection of cognitive impairments (Wesierska et al., 2005). Cognitive coordination is the ability to manage multiple conflicting information streams and selectively pay attention to relevant information while ignoring irrelevant information. A recent study (Lobellova et al., 2013) showed that active place avoidance on a Carousel in its reversal modification was more sensitive to cognitive impairment by acute dizocilpine administration than was the Morris water maze (MWM) (but for opposite cases for acquisition, see Stuchlik et al., 2004 or Vales et al., 2006). Active place avoidance with reversal is an aversively motivated dynamic-environment-task with high demands for “perceptual segregation” (cognitive coordination) and “mnemonic” segregation. Perceptual segregation is the continuous segregation of multiple frames of reference, i.e., information streams where arena- and room-frames of reference are in continuous conflict (Abdel Baki et al., 2010). Mnemonic segregation has been tested in reversal modification with the need to segregate previous irrelevant memory for to-be-avoided zone from the new one (Perera et al., 2013). As mentioned before, the role of D2-like receptors in the flexibility of spatial avoidance behavior is an understudied phenomenon, which makes this task less comparable to other studies, but at the same time is capable of providing new insights into dopamine function in learning.

Specifically, in this experiment reversal learning in QNP-sensitized drugged and undrugged rats was examined from the viewpoint of a rat model of OCD. Since acute QNP treatment induced a reversal learning deficit (Boulougouris et al., 2009), we expected to confirm such deficit would be seen after repeated QNP treatment under the drug’s effect in first experiment. In the second experiment, reversal learning was tested in sensitized but undrugged rats. Since undrugged behavior appears to mimic aspects of the behavior of sensitized drugged rats we hypothesized a reversal learning deficit would be apparent due to high sensitivity of the reversal part of the task (Lobellova et al., 2013).

## Materials and Methods

### Experimental design

Two consecutive experiments were conducted. Experiment 1 tested acquisition and reversal in QNP-sensitized rats in a drugged state during the acquisition and reversal sessions on a Carousel. Experiment 2 had the same experimental design and tested acquisition and reversal in QNP-sensitized but undrugged rats. Since these two experiments were conducted at different time periods, pooling experiments together would not be statistically correct, so they are reported separately. Experiment 1 compared learning between QNP-sensitized drugged rats with QNP (*n* = 10) and saline-treated rats (*n* = 11). Experiment 2 compared learning between QNP-sensitized undrugged rats (*n* = 10) and saline-treated rats (*n* = 9).

### Rats

Adult male Long–Evans rats from the breeding colony of the Institute of Physiology AS CR were used. All rats weighed 300–400 g at the start of experiment and were 12–15 weeks of age. Rats were housed 3–4 rats per cage in an air-conditioned rat room with a stable temperature of 22°C, constant humidity, and 12/12 light/dark cycle. Both experiments were conducted in the light phase of the day. Food and water were freely available. Prior to the experiments, rats were handled for 2 min daily for 3 days. Rats were also gently implanted with a subcutaneous needle connector, which pierced the skin between rat’s shoulders. The needle had a blunted and swirled tip for the attachment of an alligator clip connecting a shock-delivering wire. This procedure is analogous to a hypodermic injection in humans and does not require anesthesia. All rat manipulations were conducted in accordance with the Animal Protection Code of the Czech Republic and a corresponding directive of the European Community Council on the use of laboratory animals (2010/63/EC).

### Drugs

Quinpirole hydrochloride (Sigma-Aldrich, Czech Republic, Cat. No. Q102) was dissolved in saline solution (0.9% NaCl) to achieve a concentration of 0.5 mg/mL. When appropriate, each rat was injected with 1 mL/kg of QNP solution or a corresponding volume of saline solution (1 mL/kg).

### Apparatus – Carousel

**Figure 1:**
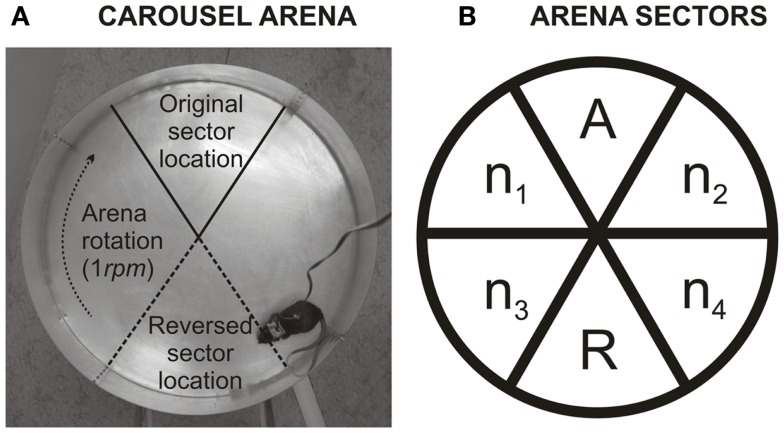
**(A)** Illustration shows the Carousel arena. The arena is a rotating metallic disk on which is, directly imperceptible by the rat, a 60° sector where it receives a mild electric shock. This sector can be localized only by using extra-maze cues, which are abundant in the experimental room. As not to disturb, the experimenter observes rat’s movement on a TV screen from a different room. **(B)** Describes calculation of *percentage of time spent in the former to-be-avoided sector*. The arena was divided into six equal non-overlapping sectors. *A* labels the former shock sector (during ACQ1–ACQ4). *R* is the segment of the arena that is punished in reversal (and was formerly safe) (REV1–REV4). *n*1–*n*4 are sectors that were never punished. Level of perseveration can be inferred from the *percentage of time spent in the former shock sector* (*A*) of time spent in safe sectors during first 10 min of reversal (*A*+*n1*–*n4*): $\%A=\left(\frac{A}{A+n1+n2+n3+n4}\right)\times 100$. High perseveration is more likely when a rat is less in the former to-be-avoided sector, while low perseveration is more likely when rat spends a higher fraction of time there. To note, 20% indicates that a rat distributes its time equally between all safe sectors.

The apparatus (Carousel, Figure 1A) is a circular metallic disk (82-cm diameter) elevated 1 m above the floor with a low rim. The arena is surrounded by 60-cm-high transparent Plexiglas wall. The arena rotated at 1 revolution/min in a clockwise direction. An unmarked 60°-to-be-avoided sector was defined in stable room-frame coordinates on the rotating arena. Whenever a rat entered the sector for more than 300 ms, constant-current regulated electric footshocks (AC, 50 Hz, 200–600 μA) were delivered at 1200-ms intervals until the rat left the sector. The shocks were administrated through the above-described subcutaneous needle connector implanted on the back of the rat standing on the grounded floor. The highest voltage drop of the current passing through the rat was at the high-impedance contact between the paws and grounded metal floor. The appropriate current was individualized for each rat in order to elicit a rapid escape reaction but prevent freezing. This aversive procedure has been shown to be efficient and safe in previous studies (for review, see Stuchlík et al., 2013).

Each rat was allowed to move freely within the arena boundaries. To localize the sector rats had to navigate purely using distant extra-maze landmarks because proximal intra-maze landmarks (such as scents, urine marks, or feces) were made irrelevant by arena rotation. During acquisition (ACQ) sessions the to-be-avoided sector was arbitrarily defined at North. During reversal (REV) sessions the sector was relocated to South – the opposite side of the disk, while the direction of arena rotation remained the same.

The constant-current-regulated source, which carries current for the shock application also contains a unit for powering a light-emitting diode (LED), attached by a latex harness on rat’s back signaling the position of the rat to an overhead camera and a computer. The second LED diode is on the arena periphery signaling arena rotation. The analog signal from an overhead infrared camera is digitized by a DT-3155 card (Data Translation, USA) in the Tracker program (Biosignal Group, USA), which samples rat’s position at the rate of 25 Hz.

### Quinpirole sensitization and habituation to Carousel

Prior to avoidance testing, rats were sensitized by repeated administration of a QNP solution (or saline solution for control groups) in the course of 3 weeks. QNP (0.5 mg/kg) was applied on Mondays, Wednesdays, and Fridays up to a total of 10 injections. Sensitization was conducted in the same Carousel apparatus where later avoidance learning was tested. During the sensitization procedure each rat received a QNP or saline injection in its home cage and 30 min later was placed onto the Carousel for 30-min exploration (with no shock). This means that sensitization was conducted together with repeated exposure to the experimental environment. To minimize potential conditioning to the injection schedule, the order of rats during sensitization was varied pseudo-randomly.

### Procedure – acquisition and reversal testing

Behavioral testing included two phases – acquisition (ACQ) and reversal (REV). Acquisition preceded reversal. Both acquisition and reversal sessions took place in four 30-min sessions each conducted every other day. The only difference in setup between acquisition and reversal sessions was the location of the sector (180° shift).

**Figure 2:**
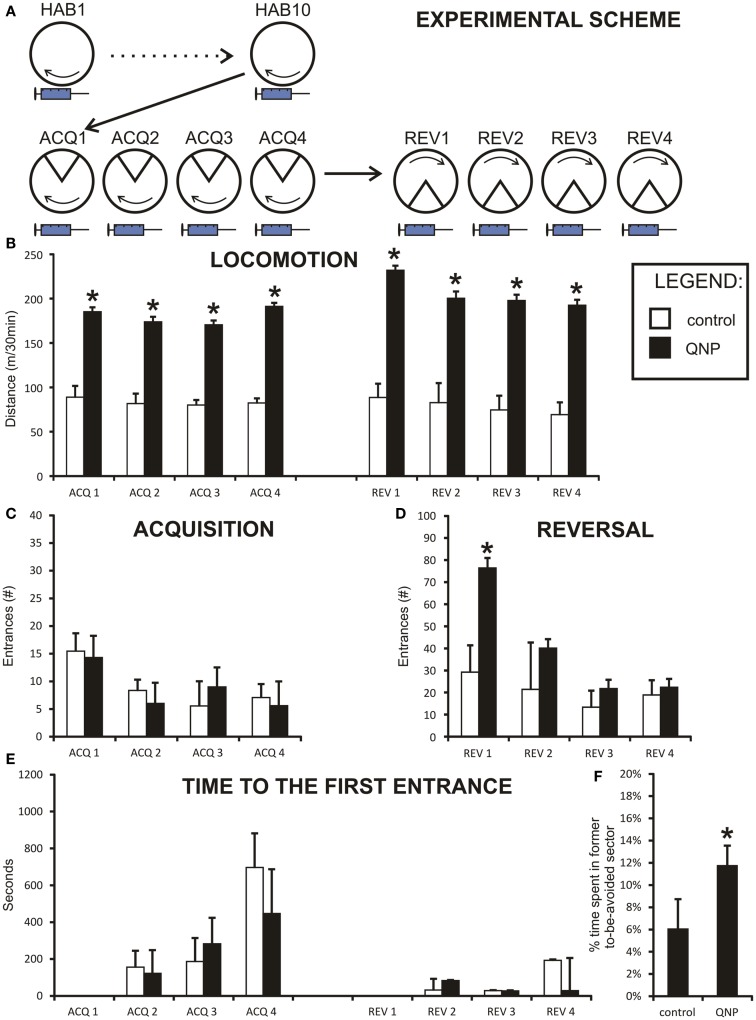
**Graph shows results from experiment 1 (QNP-sensitized drugged rats)**. Data are presented as mean values ± SEM. Asterisks denote significant simple effect analysis that followed significant group effect in ANOVA (*p* < 0.05). **(A)** The scheme of experiment 1. **(B)** Locomotion (meters/30 min) of chronically treated drugged with QNP-rats compared to saline-treated rats. There is significantly higher locomotion in QNP-rats compared to their controls during all sessions (ACQ1–REV4). **(C)** Number of entrances into the to-be-avoided sector (#/30 min) during acquisition sessions (ACQ1–ACQ4) when all rats are included. **(D)** Number of errors (#/30 min) in four reversal sessions (REV1–REV4). Data show a significant difference in number of errors in first day of reversal testing (REV1). **(E)** Time to the first error (mean seconds ± SEM) in experiment 1 (ACQ2–ACQ4; REV2–REV4). There was no significant effect at *p* < 0.05. **(F)** *Percentage of time spent in former shock sector* (*A*) compared to mean time spent in always safe sectors (*A* + *n*1–*n*4) during first 10 min of reversal (REV1). QNP-treated group shows significantly higher percentage of time spent in former to-be-avoided sector, indicating more distributed time spent in all safe sectors, suggesting lower rate of perseveration than control rats.

**Figure 3:**
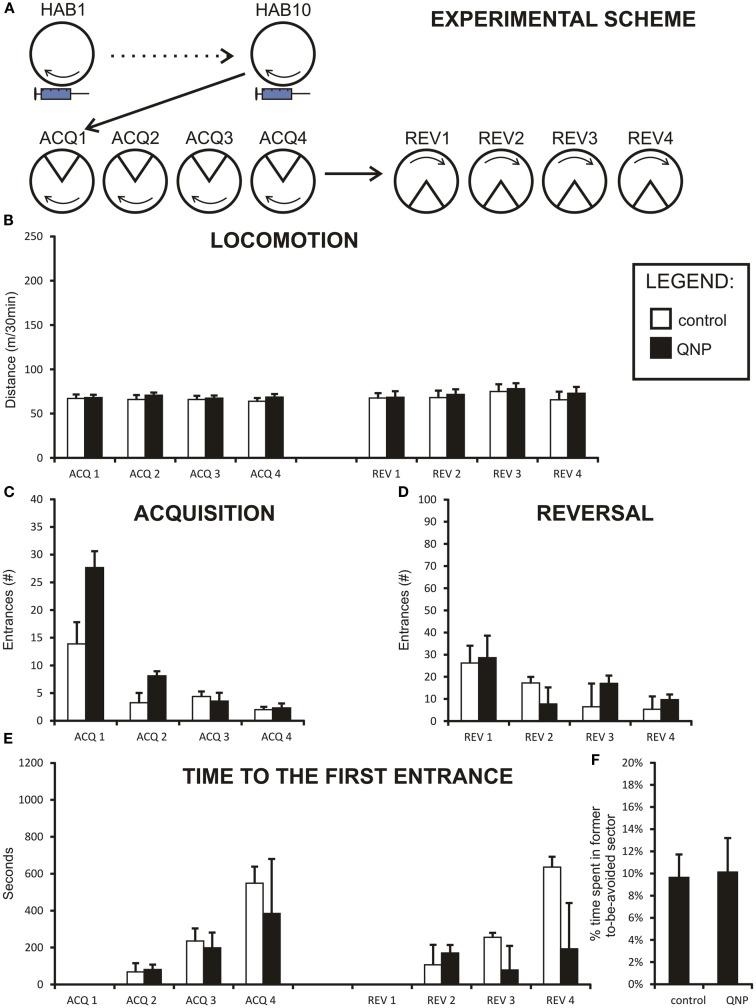
**Graphs illustrating results from experiment 2 (QNP-sensitized but undrugged rats)**. Data are presented as mean values ± SEM. Asterisks denote significant simple effect analysis that followed significant group effect in ANOVA (*p* < 0.05). **(A)** The scheme of experiment 2. **(B)** Locomotion (meters/30 min) of sensitized rats. It is apparent that there is no significant difference in locomotion between QNP-sensitized rats and a non-sensitized control rats. **(C)** Number of errors (#/30 min) during all acquisition sessions (ACQ1–ACQ4). **(D)** Number of errors during four reversal sessions (#/30 min; REV1–REV4). Significant interaction term is not displayed, but data suggest that it is due to higher error rate in QNP-sensitized rats (REV1) compensated by faster learning in a second reversal day (REV2). **(E)** Time to the first error in seconds (mean ± SEM) in experiment 2 (ACQ2–ACQ4; REV2–REV4). There was no significant effect at *p* < 0.05. **(F)** *Percentage of time spent in former shock sector* (*A*) compared to mean time spent in always safe sectors (*A* + *n*1–*n*4) during first 10 min of reversal (REV1). QNP-sensitized rats appear not to differ from non-sensitized control rats in distribution of movement in safe sectors of the arena (and avoidance of previously punished sector).

In both experiments (1 and 2) testing in the Carousel commenced 2 days after sensitization/habituation sessions ended (10 sessions, see Figures 2A and 3A for experimental scheme illustrations). Rats in experiment 1 received an injection of 0.5 mg/kg QNP or saline 30-min prior to the arena testing. Rats in experiment 2 were not treated at all during the testing and therefore did not require any time delay before placement into the arena. In the beginning of each session, each rat was placed into the arena opposite to the location of the shock sector, facing the experimenter. Carousel rotation and tracking was turned on immediately after an experimenter left the room. Since the arena was rotating independently of the to-be-avoided sector, the best strategy to solve the task was to walk constantly or intermittently in the counter-clockwise direction to avoid being transported into the shock sector by arena rotation. Our observations suggest that four acquisition and four reversal sessions are sufficient for rat to acquire a successful learning strategy.

### Measured parameters and statistical analysis

Parameters presented here were extracted from an offline analysis program for Tracker (Track Analysis, Biosignal Group, USA) and an open-source Carousel Maze Manager (Bahník, 2013). The output parameters that were assessed in analysis were locomotor activity measured as distance walked throughout a session in meters (movement of arena detected by peripheral LED diode was subtracted from total locomotion), number of entrances into the sector (errors), time to the first error, and *percentage of time spent in the former to-be-avoided sector* during reversal.

First, we assessed a distance animals walked during the session because locomotion between experimental groups was expected to differ due to the stimulant effect of QNP. If the expected locomotor activity difference between control and treatment groups in experiment 1 was present, an issue of hyperlocomotion influencing the number of errors had to be addressed. Hypothetically, if a rat walked randomly into the arena, higher locomotor activity would result in an increased number of errors per unit of time into the shock sector solely by chance. To assure that the variation in number of errors between the groups was not associated with higher locomotion in QNP group; correlations were computed to assess a relationship between these two parameters. For all correlation analyses when data showed normal distribution Pearson’s product moment coefficient was used and when data were not normally distributed Spearman’s rho was used.

The principal spatial parameter was the *number of errors*. A low number of errors reflect comprehension of the task, well-managed avoidance strategy, and intact cognitive coordination. Additionally, to assess long-term between-session memory, *time-to-first-error* was analyzed. Since a rat was never placed directly into the sector, a solid memory trace enables the rat to avoid receiving a shock from the very beginning of every session with the exception of the first day of acquisition (ACQ1) and first day of reversal (REV1).

To discern types of errors that animals make when the to-be-avoided sector is reversed, *percentage of time spent in former to-be-avoided sector* was assessed for the first day of reversal (REV1). The calculation is similar to one described in detail by Petrasek et al. (2013). In short, the arena was divided into the six 60°-sectors. One of these sectors was the one reinforced in acquisition sessions (now a former to-be-avoided sector; *A*), the second sector was a current (reversed; *R*) sector (opposite to former to-be-avoided sector). The remaining four sectors, *n*1–*n*4, were never punished and were located in pairs between former and currently to-be-avoided sector (illustrated in Figure 1B). The percentage was calculated from a ratio of time spent in the former to-be-avoided sector divided by average time spent in always safe sectors [*A/*(*n*1–*n*4 + *A*)]. The reversed (current) to-be-avoided sector was excluded from the calculation because electric shock affects the time spent in this sector. Since perseveration can be quickly overridden by re-learning, the new sector position, the initial 10 min of the first reversal session were analyzed. In the reversal learning task, rats can make two types of errors. One type of mistake can be produced by the inability to shift from the previously relevant strategy – referred to as perseverative errors. Other types of mistakes result from the inability of the rat to learn a new strategy – referred to as memory saturation. A lower percentage indicates, with a high incidence of errors, that the rat failed because it avoided former to-be-avoided sector during reversal learning, while failing to adapt to the new shock location. The higher percentage (ratio) (20% indicates an equal preference of all five safe sectors) suggests that the rat avoided former shock sector less, which could indicate either low perseveration or weak long-term memory. If a high number of errors would accompany high ratio, it can be claimed that these errors were not perseverative in nature but caused by other factors such as memory saturation.

Every batch of rats used in this study included rats which did not learn the paradigm. An inability to achieve effective avoidance can be caused by many factors often unrelated to the experiment itself, such as breeding issues or an unknown stressful event. Specific cases are rats which do not opt for avoidance strategy but instead display prolonged freezing resulting in a complete absence of avoidance behavior and locomotor activity (approximately 10% of all rats, unpublished observations from multiple experiments). These rats and rats which did not find effective learning strategy in acquisition had to be excluded from the reversal learning task (A rat cannot learn to reverse the task if it did not learn it in the first place.). For the exclusion of rats in the reversal a threshold of minimum 10 errors during the last 30-min session was used (ACQ4).

For the assessment of learning differences in acquisition and reversal sessions, a two-way repeated measures ANOVA was conducted using the factor of sessions as a repeated measure (session; ACQ1–ACQ4, REV1–REV4) and groups as a between-subject factor (QNP vs. saline). Significant ANOVA was followed by simple effect analysis when sessions × groups interaction was significant. If necessary, acquisition learning was analyzed twice, once with included and once with the excluded “non-learners.” This is to uncover any bias that could be present due to exclusion of non-learning rats (i.e., non-learners had greater impact in one group than in the other). If the data were not normally distributed or did not meet the assumption of homogeneity of variance, appropriate transformations (logarithmic; for acquisition sessions in experiment 1, acquisition and reversal in experiment 2) were conducted. If no transformation was able to transform data into the parametric data sets, differences between the groups were assessed by a non-parametric Mann–Whitney sum ranks test with Bonferroni correction applied to the level of test significance (acquisition with non-learners included in experiment 1). All statistical tests were considered significant at the threshold of *p* < 0.05 (two tailed).

## Results

### Experiment 1

This experiment assessed learning in rats sensitized with dopamine D2-like agonist QNP under QNP treatment (Figure 2A). Two rats in each group did not reach learning criterion of having <10 errors in the last acquisition session (QNP group: rats with 17 and 21 errors; control group: rats with 30 and 37 errors). These rats were not included in reversal learning. Specifically, in the control group one of these rats froze throughout most of the session and second did not find an effective avoidance strategy. In QNP group neither of the two excluded rats appeared to abide by an effective avoidance strategy (visual observation).

### Locomotion

All rats from experiment 1 were included in the assessment of locomotor activity throughout all four acquisition and four reversal sessions by repeated measure two-way ANOVA. Data were normally distributed in both groups, and variances between groups were not significantly different. Because Mauchly’s test indicated that the assumption of sphericity had been violated, [χ^2^(27) = 66.99, *p* < 0.001], degrees of freedom were corrected using the Greenhouse–Geisser estimate of sphericity (ε = 0.45) for tests that included a repeated measure. There was a significant main effect of sessions on distance [*F*(3.26, 52.15) = 2.95, *p* < 0.05] and significant effect of groups [*F*(1,16) = 234.05, *p* < 0.001]. Also, analysis showed a significant effect of sessions × groups interaction [*F*(3.26, 52.15) = 3.78, *p* < 0.05]. Planned contrasts for the effect of both sessions and interaction term showed a significant change in locomotion between acquisition day 3 (ACQ3) and day 4 (ACQ4) [session: *F*(1,16) = 9.69, *p* < 0.01; interaction: *F*(1,16) = 9.17, *p* < 0.01] and between acquisition day 4 (ACQ4) and first day of reversal (REV1) [session: *F*(1,16) = 11.25, *p* < 0.01; interaction: *F*(1,16) = 9.49, *p* < 0.01]. Because by visual inspection control group did not show any fluctuations (Figure 2B) in locomotor activity, the up-regulation of activity in the QNP group probably accounts for both significant sessions and interaction effect.

### Distance-errors correlation

**Figure 4:**
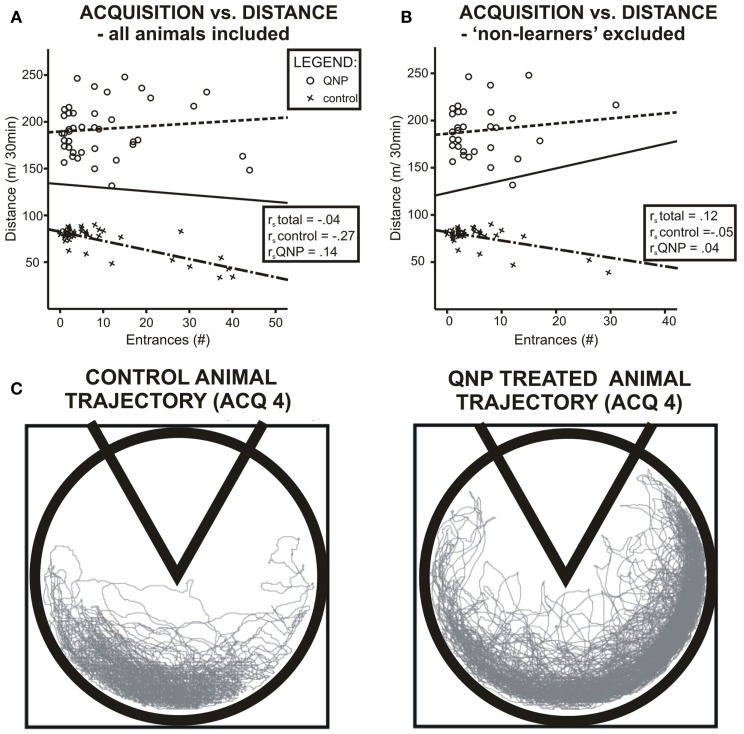
**Scatter plots visualizing correlation between number of errors and locomotion in experiment 1**. **(A)** Data when all rats are included. **(B)** Only rats that will enter reversal (non-learners excluded). Blue color denotes QNP-treated rats, green color saline-treated rats. Both groups show different patterns of association between number of errors and distance walked, namely in the QNP group, there is a trend of worsened performance (more errors) with higher locomotion, and in the saline-treated group, there is an opposite trend. Nonetheless, this trend was not significant with or without non-learners included. **(C)** Trajectory of hyperlocomotor QNP-treated and control rat where neither individual made any error into the to-be-avoided sector. Control and QNP-sensitized and drugged rats’ trajectories are from last-day of acquisition (ACQ4).

When groups were pooled together, (including excluded rats) data did not show any correlation between distance walked and errors during all acquisition sessions pooled together rs = −0.04, ns. However, it is obvious from the graph (Figure 4A) that data were sectioned into two distinct populations based on treatment a rat received. When treatment groups were considered separately there was no significant correlation between number of errors and locomotor activity, (rs = −0.266, ns), in control group and no statistically significant correlation in the QNP-treated group, (rs = 0.136, ns). After exclusion of rats that did not reach the threshold of learning there was still no overall or group specific significant correlation between number of errors and distance walked by a rat (control group: rs = −0.05, ns; QNP group: rs = 0.04, ns; pooled groups: rs = 0.12, ns) (Figure 4B). This indicates that in rats which have entered reversal testing, there was no correlation between the distance and number of errors despite the large difference in locomotion between the groups. To visually compare trajectories of hyperlocomotor QNP-treated and control rats, example trajectories of each of these rats are included where individuals did not make any entrance into the to-be-avoided sector. Figure 4C depicts an example in ACQ4 of the trajectories of locomotion for a control rat and for a QNP-sensitized rat under QNP.

### Acquisition and reversal learning

Learning behavior was analyzed for acquisition and reversal learning separately. Acquisition learning (ACQ1–ACQ4) was analyzed with all cases included (to determine if there is a significant difference in overall learning capability between groups) and with “non-learners” excluded from analysis.

In acquisition learning, when all rats were included, the number of errors distributions was not normal and no transformation was able to normalize them. Therefore, the Mann–Whitney test was used to compare two experimental groups in each acquisition learning day. The Bonferroni correction was applied to control for family wise error caused by the high number of comparisons (new significance threshold was calculated to be *p* < 0.0125). No significant differences were detected between groups in any of the acquisition sessions (ACQ1: *U* = 42.00, *z* = −0.92, ns; ACQ2: *U* = 54.50, *z* = −0.04, ns; ACQ3: *U* = 41.50, *z* = −0.97, ns; ACQ4: *U* = 42.50, *z* = −0.90, ns) (Figure 2C). The results show there was no difference between QNP and control groups in acquisition learning with all rats included in the study.

After the exclusion of rats which did not meet learning criteria (therefore only rats, which learned acquisition task remained) data showed normal distribution and equal variances after logarithmic transformations, which allowed two-way repeated measure ANOVA to be conducted. Because Mauchly’s test indicated that the assumption of sphericity had been violated [χ^2^(5) = 42.39, *p* < 0.001], degrees of freedom were corrected using the Greenhouse–Geisser estimate of sphericity (ε = 0.40) for tests that included repeated measure. Session was considered a repeated measure and group was a between-subject factor. The only significant effect was main effect of session [*F*(1.19,17.88) = 26.59, *p* < 0.001]. There was no significant group effect [*F*(1,15) = 39.95, ns] or sessions × groups interaction [*F*(1.19,17.88) = 0.93, ns]. No simple effect *post hoc* analysis was conducted since there were no significant differences between the groups (data not shown).

Two-way repeated measure ANOVA was conducted on the number of errors in four reversal sessions (REV1–REV4, only rats which achieved <10 errors in ACQ4 were included) (Figure 2D). Data were normally distributed so no transformation was needed. Assumption of homogeneity of variances was broken and it was not possible to correct it with any transformation. Therefore, the Brown and Forsythe correction was applied during *F* score calculation. Specifically, effect of session was significant [*F*(1.939, 25.204) = 5.444, *p* < 0.05], even after degrees of freedom were corrected by the Greenhouse–Geisser estimate of sphericity (ε = 0.65) because the assumption of sphericity was significantly violated [χ^2^(5) = 18.673, *p* < 0.05]. Sessions × groups interaction was also significant, even after the Greenhouse–Geisser correction to degrees of freedom [*F*(1.94, 25.20) = 4.61, *p* < 0.05]. Effect of groups was also highly significant [*F*(1,13) = 31.72, *p* < 0.001]. To break down an interaction term between QNP-treated and control rats, simple effect analysis was conducted. It showed that a significant difference was observed only on the first day of reversal (REV1) *F*(1,13) = 11.18, *p* < 0.01 [other sessions: REV2 *F*(1,13) = 2.14, ns; REV3 *F*(1,13) = 0.73, ns; REV4 *F*(1,13) = 0.02, ns].

### Time to the first error

Between-session long-term memory was measured by time-to-the-first error. Data were non-parametrically distributed and no transformation was capable of normalizing them. Therefore, the non-parametric Mann–Whitney test had to be used to analyze the data. The Bonferroni correction was applied to control for family wise error caused by high number of comparisons (new significance threshold was calculated to be *p* < 0.008). Despite the apparent trend (Figure 2E) we did not find the differences in this parameter significant (ACQ2: *U* = 53.00, *z* = −0.14, ns; ACQ3: *U* = 37.00, *z* = −1.27, ns; ACQ4: *U* = 27.00, *z* = −1.97, *p* = 0.049; REV2: *U* = 52.00, *z* = −0.21, ns; REV3: *U* = 44.50, *z* = −0.42, ns; REV4: *U* = 49.00, *z* = −0.42, ns). In summary, after the family wise correction of significance threshold, there was no significant difference in between-session memory between saline-treated rats and rats chronically drugged with QNP.

### Percentage of time spent in former to-be-avoided sector during reversal

A more detailed look into the differences in reversal learning behavior is offered by analysis of time spent in the former to-be-avoided sector after change of shock location. It is defined by percentage of time spent in the formerly to-be-avoided sector of time spent in always safe sector (all sectors excluding the reversed to-be-avoided sector). Intriguingly, *t*-test shows that control rats spent only 5.12% (SEM = ±1.7%) of the time in former shock zone while QNP-treated rats spent up to 13.32% (SEM = ±2.5%) in the former shock zone in the first 10 min of first reversal session (REV1) [*t*(15) = −2.76, *p* < 0.05] (Figure 2F). On a probability basis it could be argued that with a higher locomotion rate QNP-treated rats enter the former shock zone sooner and more often, an experience which could quickly dis-inhibit the previously learned response. Therefore a correlation analysis was conducted to assess any relationship between distance and measure of perseveration. The correlation analysis showed that there was no significant correlation between the two measures in this study (rs = 0.31, ns). In conclusion, QNP-treated rats entered former-to-be-avoided sector significantly more than control rats. These results do not show perseverative behavior in controls and demonstrate that QNP-sensitization and treatment does not increase perseveration during the reversal task on the Carousel.

### Experiment 2

This experiment aimed to assess an effect of sensitization by dopamine D2-like agonist QNP on acquisition and reversal learning on a Carousel in undrugged rats (for experimental scheme see Figure 3A). Three rats were excluded from the analysis due to technical complications (one from QNP-treated group and two control rats). Rats which remained reached a pre-defined threshold of max 10 errors by the fourth acquisition sitting (ACQ4). Thus eight control rats and nine QNP-treated rats were included in all analyses.

### Locomotion

Two-way repeated measure ANOVA was conducted to compare the locomotion between group sensitized to QNP (but undrugged during both acquisition and reversal learning sessions) compared to control rats. Data were normally distributed and variances of the groups were not significantly different. Mauchly’s test indicated that an assumption of sphericity for repeated measure test had not been met [χ^2^(27) = 65.56, *p* < 0.001] and therefore all the degrees of freedom in repeated measure tests (session and sessions × groups interaction) were corrected by a Greenhouse–Geisser estimate of sphericity (ε = 0.40). As can be observed from the graph (Figure 3B) there was no group effect in locomotion between sensitized and control rats [*F*(1,15) = 0.004, ns]. The locomotion remained stable throughout sessions for there was no effect of session observed [*F*(2.77,41.57) = 0.75, ns]. Non-significant sessions × groups interaction terms indicate that the two groups did not differ with the regard to locomotor activity [*F*(2.77,41.56) = 0.197, ns.].

### Acquisition and reversal learning

Acquisition and reversal learning was analyzed separately and all rats were included in the study. Since locomotion between the groups did not vary, there was no need for correlation analysis testing the relationship between distance and number of errors into the to-be-avoided sector. In both acquisition and reversal learning, the *number of errors* data were not normally distributed and had to be transformed by a logarithmic transformation to correct the issue. Variances between the groups were homogenous.

Two-way ANOVA was conducted to compare acquisition learning between QNP-sensitized and control rats (Figure 3C). The effect of session [*F*(2.05,30.72) = 50.68, *p* < 0.001] was significant even after the Greenhouse–Geissler correction, of sphericity (ε = 0.68) which was necessary because the data significantly deviated from the assumption of sphericity [χ^2^(5) = 13.04, *p* < 0.05]. There was no significant group effect observed [*F*(1,15) = 3.36, ns], or sessions × group interaction [*F*(2.05, 30.72) = 1.59, ns]. The results suggest that in acquisition learning there were no differences in learning between QNP-sensitized and control groups (Figure 3B). Although interaction term was not significant from the inspection of the graph it appears that there is a difference in performance on ACQ1 where QNP-rats performed worse than control rats (more errors).

Reversal session learning measured by number of errors was also analyzed using two-way repeated measure ANOVA. Data were logarithmically transformed to correct for deviation from normal distribution. Since the sphericity assumption was met, no adjustment to degrees of freedom was necessary in results which included repeated measure. The effect of session was highly significant [*F*(3,45) = 12.72, *p* < 0.001], while group effect was shown to be not significant [*F*(1,15) = 0.18, ns]. Significant interaction term sessions × group [*F*(3,45) = 3.64, *p* < 0.5] indicated that there was a difference in learning between the QNP group compared to the control group (Figure 3D). Nonetheless, the interpretation of this effect must be very cautious because there was no observable group effect in reversal. We checked if the significant interaction could not have been caused by one or two highly deviating animals. After Grubb’s test to detect outliers it was found that one rat in the QNP group was a significant outlier. However, even after removal of this outlying point, the interaction effect remained significant [*F*(3,42) = 3.11, *p* < 0.05] (assumption of sphericity was met). By visual observation, it appears that QNP-treated rats learn significantly faster than control rats between REV1 and REV2. In summary, in experiment 2 there was no effect in acquisition learning between QNP-sensitized and control rats, but a significant interaction term may suggest an initial steeper learning curve in QNP-treated rats in reversal learning.

### Time to the first error

Time-to-first-error was analyzed by a non-parametric Mann–Whitney test since no transformation was capable of normalizing the data. A Bonferroni correction was applied to control for family wise error caused by a high number of comparisons (new significance threshold was calculated to be *p* < 0.008). First day of acquisition (ACQ1) and first day of reversal (REV1) were excluded from the analysis since time-to-first-error measures a memory trace could not be present at the beginning of these two sessions. No significant differences were detected between groups in any of the acquisition days (ACQ2: *U* = 26.00, *z* = −0.96, ns; ACQ3: *U* = 32.00, *z* = −0.39, ns; ACQ4: *U* = 30.00, *z* = −0.58, ns; REV2: *U* = 31.00, *z* = −0.48, ns; REV3: *U* = 22.00, *z* = −1.35, ns; REV4: *U* = 35.00, *z* = −0.10, ns) (Figure 3E). Thus, our analysis did not find any difference in the long-term between-the-session memory in rats sensitized to QNP.

### Percentage of time spent in former to-be-avoided sector during reversal

*Percentage of time spent in the former to-be-avoided sector* was calculated for experiment 2 in the same manner as for experiment 1. A *t*-test was used to compare the difference in this value between groups. Results showed that there was no difference in percentage of time spent in the former to-be-avoided sector out of all safe sectors between control and QNP-sensitized groups [*t*(15) = 0.11, ns] (Figure 3F) with the mean percentage spent in the former shock sector being 9.9% (SEM = ± 2.8%) and 9.52% (SEM = ± 2.1%), respectively.

## Discussion

Quinpirole-sensitized drugged rats during cognitive testing (experiment 1) showed comparable acquisition learning with control rats but displayed impaired reversal learning, which was not associated with perseverative responding. Rats acquired the task at a similar rate as the control group, despite hyperlocomotion in QNP-drugged rats, and the same number of rats per group reached the threshold of 10 errors in 30-min session by the fourth session. This indicated that chronic sensitization of dopamine D2-like receptors by QNP (and their ongoing stimulation) did not affect cognitive coordination (perceptual segregation). Cognitive coordination deficits are consistently observed in schizophrenia patients (Han et al., 2012) and in rat models of schizophrenia (Lobellova et al., 2013). The present lack of effect on cognitive coordination suggests that chronic QNP treatment did not involve such an aspect. It can be speculated that defective acquisition learning due to the effect of impaired cognitive coordination in schizophrenic patients (Phillips and Silverstein, 2003) was not caused by sensitized dopamine D2-like receptors despite clear evidence of dopamine involvement in the pathology of schizophrenia (Carlsson et al., 1999). It is very interesting that despite much higher locomotion rate, these animals managed to avoid to-be-avoided sector with no problem. However, dispersion of locomotion in the safe part of the arena is much wider in QNP-treated animals. This may be related to the hyperlocomotion induced by QNP.

In the reversal learning, QNP-sensitized drugged rats showed a significant, but transient reversal deficit manifested by an increased number of errors during the first session compared to the control group. It should be noted that this deficit was indeed specific only for the beginning of reversal training, since by the third and fourth reversal session the deficit was ameliorated and rats displayed comparable results with the control group.

It was proposed that there are three parallel processes that have to occur during successful reversal: extinction of response that is no longer rewarded, behavioral switch to the new reward, and response maintenance (Klanker et al., 2013). A deficit in extinction would be characterized by a perseverative responding. A defect in behavioral switch would be associated with disorganized behavior while a defect in response maintenance would be associated with the inability to improve in both acquisition and reversal tasks (both between sessions and within one session). Our results suggest that the only defective process in the case of QNP-sensitized drugged rats is the behavioral switch. From the results, it is apparent that in reversal, control rats did not significantly improve their performance, compared to improvement observed in QNP-treated rats. This could indicate that QNP actually improves response maintenance. However, since significant improvement was observed in control group in experiment 2, which received exactly the same treatment, this effect might also be a batch-specific artifact.

Importantly, the observed reversal deficit was not associated with the perseverative behavior as can be deduced from *percentage of time spent in the former to-be-avoided sector*. QNP-treated rats actually spent a higher percent of time in the former to-be-avoided sector than control rats. We hypothesized that QNP-treated rats would perseverate – to keep avoiding the former to-be-avoided sector – more than controls based on studies that had shown that chronic administration of QNP is associated with perseverative behavior in alternation tasks (Einat and Szechtman, 1995; Kontis et al., 2008) and on studies that document enhanced “compulsive” lever pressing after repeated administration of QNP (Joel et al., 2001). Also, the only study that tested reversal in rats treated with systemic acute QNP reported a marked reversal learning deficit associated with high incidence of perseverative responding (Boulougouris et al., 2009). Despite the often observed perseverative behavior, non-perseverative behavior in reversal was also reported following D2-like manipulation. For example, non-perseverative errors in reversal were demonstrated when QNP was infused locally into the nucleus accumbens (Haluk and Floresco, 2009) and after dopamine depletion in the *striatum* (Clarke et al., 2011) or depletion in orbitofrontal cortex (Walker et al., 2009). Non-perseverative errors were observed in reversal learning in humans on spatial tasks after systemic administration of bromocriptine, another D2-like agonist (Mehta et al., 2001). Although not uncommon, a lower perseveration in QNP-treated rats in our study is very intriguing in light of previous studies specifically regarding the effects of systemic QNP administration, which showed high perseverative behavior in QNP-treated rats (Kontis et al., 2008; Boulougouris et al., 2009).

We propose several theories to explain why QNP-treated rats were entering faster into the former to-be-avoided sector than control rats despite often cited increased perseveration during the reversal task. Possibly, drugged rats sensitized to QNP might have exacerbated checking, which would suggest that they might have inspected the previously punished region more frequently. Also, fast entrance into the former to-be-avoided sector may be simply caused by an increased locomotion – as mentioned before, animal that moves more has a higher chance of entering into any location of the arena sooner. Alternatively, QNP-treated animals could simply enter the former to-be-avoided sector because of impaired long-term memory. This hypothesis is supported by the trend we observed in time-to-the-first-error. Although not significant, QNP-treated animals consistently entered to-be-avoided sector very soon after session commencement, suggesting these animals had to be “reminded” where the to-be-avoided sector is located. It is interesting that in our experiments the trend of disruption of between-session learning is observed in QNP-sensitized animals regardless of drugged state (in both experiments 1 and 2). Also, in OCD patients a long-term episodic memory appears to be impaired (Savage et al., 2000; Deckersbach et al., 2004). Despite the results appearing very suggestive, they never reach a significant level presumably due to high variability within the control group. A larger cohort of animals would be necessary to properly address the issue.

In human studies, rather ambiguous evidence of a reversal learning deficit was demonstrated in OCD patients. Alterations in fronto-striatal circuits (without reversal learning deficit *per se*) were observed during reversal in OCD patients (Remijnse et al., 2006) and their unaffected relatives (Chamberlain, 2007). Some studies detected worsened overall performance on the reversal task (Remijnse et al., 2006) but most found only increased time latencies to complete the task possibly indicating increased cognitive demand (Valerius et al., 2008; Remijnse et al., 2009; Ersche et al., 2011). It must be noted that in active place avoidance on a Carousel, time is an important limiting factor. A rat does not have infinite amount of time to make a correct choice due to the rotation of arena. Therefore, a longer latency to make a choice results in punishment (rat would be transported to the sector by arena rotation). In this light increased time latencies to make a choice observed in patients could be viewed as an indiscernible reversal error. Also, it was proposed that currently used reversal tasks are too simple to make gross behavioral abnormalities apparent (Klanker et al., 2013).

How cognitive flexibility is related to repetitive behavior is a relatively unaddressed topic. This is the first study, to our knowledge, to address cognitive coordination and flexibility in a QNP-induced rat model of OCD and a second study that addresses spatial flexibility in any rat model of OCD as well. Previously, only stereotyped jumping was correlated with reversal learning in a T-maze in deer mice, a genetic model of OCD, where a positive relationship between stereotypy and reversal errors was discovered (Tanimura et al., 2008). Still more studies exploring links between OCD-like behavior and reversal deficit are needed to disambiguate these somewhat contradictory findings.

An important limitation of these findings is that even acute administration of QNP is associated with reversal learning deficit; therefore the reversal learning deficit cannot be attributed solely to the model of OCD. The second limitation is a possibility of QNP-treated animals being less sensitive to electric shock, which was not directly tested. Since acquisition in both QNP and saline-treated animals is similar it can be assumed that sensitivity to electrical stimulation is unchanged by QNP treatment. Scarce literature on the QNP effect on pain sensitivity offers contradictory results with some studies proposing hyper-analgesic (Roane and Paul, 1992) and some hypo-analgesic effects (Magnusson and Fisher, 2000; Munro, 2007). Lastly, a lesion of the *nigrostriatal* dopaminergic projection had no effect on escape learning and response to electric shock suggesting that intact dopaminergic transmission is not necessary for avoidance learning (Price and Fibiger, 1975).

The second experiment (experiment 2) explored the effect of long-term sensitization by D2-like agonist QNP on reversal learning in QNP-sensitized undrugged rats. In both phases of the task rats were improving with each consecutive session. There was no difference between the groups in learning although there was worse performance in the QNP-sensitized group at the beginning of reversal. Significant interaction term in reversal learning was detected, which suggests that QNP-treated rats learn faster compared to control rats once they comprehend the task in the initial reversal session. However, this interaction term should be regarded with a caution, because of the difficulty of its interpretation. With regard to stimulants, it is known that rats remain sensitized to the substance up to a year when presented with a substance challenge (Paulson et al., 1991). As mentioned before, in QNP-treated rats perseverative behavior was observed even 12 weeks after treatment discontinuation (Einat and Szechtman, 1993). In a verification experiment, we re-applied QNP for a month and a half after discontinuation of QNP treatment, which resulted in a heightened locomotor response in these rats compared to the drug-naïve rats (unpublished results). It can be concluded, that the molecular substrate of sensitization was present at the time our experiments were conducted.

To our knowledge only one study has focused on the effect of long-term QNP administration on behavioral flexibility after it was discontinued (Einat and Szechtman, 1993). In our study we have chosen a different and, in some aspects, more demanding cognitive task – active place avoidance on a Carousel. Similarly to the previous study, we did not find any significant difference in acquisition or reversal learning between the groups [despite the time window from the last QNP treatment day was minimized to 2 days compared 10 days in the MWM experiment by Einat and Szechtman (1993)]. Also, we did not find a difference in *the percentage of time spent in the former to-be-avoided sector*, indicating lack of difference in the tendency to perseverate. This is in contrast to previously observed higher perseveration compared to saline-treated rats in MWM (Einat and Szechtman, 1993). The discrepancy could be caused by differences in the task setup, where we assessed perseveration (deduced from *percentage of time spent in the former to-be-avoided sector*) in the reversal phase. In the MWM, task perseveration was measured in the extinction session before reversal. Overall, it appears that QNP-induced sensitization is associated with alterations in reversal learning characterized by a higher error rate in the initial reversal session.

## Conclusion

We have shown a cognitive flexibility deficit in a rat model of OCD, which was not associated with increased perseveration. This robust deficit is present only when D2-like receptors are directly stimulated with QNP. When D2-like receptors are sensitized, but unstimulated by the agonist, there is no difference in number of errors between the groups in reversal of active place avoidance on a Carousel. Nonetheless, sensitized rats displayed a significantly altered learning style characterized by a higher error rate at the beginning of the reversal and faster learning in the second reversal session compared to control rats.

## Conflict of Interest Statement

The authors declare that the research was conducted in the absence of any commercial or financial relationships that could be construed as a potential conflict of interest.
